# 

**Published:** 1900-04-28

**Authors:** 


					** VW4 M. M 3T27T
a 1.1. MX mm VWi X i JO.JU*
J.jhtf 57gggBTg CI ftUHty A-Lancef. april smth. iwo.
CADBURY'S If IS* CADBURY'S
rnnna \1lV /A\ As Jl^ . COCOA
COCOA ak <MBL> COCOA
ABSOLUTELY PURS. ^ NO DRUGS OR ALKALIES USED.
No. 709. Vol. XXVIII. Saturday,
April 28, 1900.
[SutseripMon TcrmSjJ pRICE TWOPENCE.

				

